# EHMTI-0335. Depression and anxiety symptoms in patients suffering from new daily persistent headache. A prospective, controlled study

**DOI:** 10.1186/1129-2377-15-S1-C15

**Published:** 2014-09-18

**Authors:** C Deligianni, DD Mitsikostas

**Affiliations:** 1 Neurological Department, Athens Naval Hospital, Athens, Greece

## Introduction

New Daily Persistent Headache (NDPH) is a chronic primary headache with uncertain outcome. Comorbities may play important role in choosing the right treatment in order to improve the management of this disorder.

## Aim

To investigate the comorbidity of depression or anxiety of the patients suffering from NDPH.

## Methods

One hundred and forty four (144) headache outpatients (62 men and 82 women) and one hundred and fifty (150) healthy controls (56 men and 94 women) were screened prospectively using a questionnaire that included the Hamilton scales for both Anxiety and Depression.

## Results

The mean scores of both HAM-A and HAM-D scales were significantly higher in NDPH patients (19±7.3 and 16±6.3 respectively) comparing with the healthy population (6.8±4.0 and 5.7±3.3 respectively, p<0.05 for both comparisons). No significant difference was detected in the Hamilton scaling scores between NDPH and Chronic Tension Type Headache sufferers.

## Conclusion

NDPH sufferers showed more depression or anxiety symptoms than healthy controls, as headache sufferers from chronic TTH. Treating properly psychiatric comorbities in NDPH sufferers, or choosing drugs that influence both conditions simultaneously may improve outcome.

No conflict of interest.

